# Effect of vestibular rehabilitation therapy in patients with persistent postural perceptual dizziness: a systematic review and meta-analysis

**DOI:** 10.3389/fneur.2025.1599201

**Published:** 2025-09-24

**Authors:** Yanyan Li, Xiaorui Pei, Rui Ding, Ziqi Liu, Ying Xu, Zhicheng Wang, Yanmei Li, Lianhe Li

**Affiliations:** 1 Department of Neurology Ward, Chaoyang Central Hospital of China Medical University, Chaoyang, Liaoning, China; 2 Department of Laboratory of Molecular Biology, Chaoyang Central Hospital of China Medical University, Chaoyang, Liaoning, China; 3 Clinical Medicine Major, Xinhua Clinical College, Dalian University, Dalian, Liaoning, China; 4 Department of Head and Neck Surgery Ward, Chaoyang Central Hospital of China Medical University, Chaoyang, Liaoning, China

**Keywords:** vestibular rehabilitation therapy (VRT), virtual reality therapy, persistent postural-perceptual dizziness (PPPD), dizziness handicap inventory (DHI), vestibular rehabilitation

## Abstract

**Background:**

Persistent Postural-Perceptual Dizziness (PPPD) is an increasingly recognized syndrome defined by enduring experiences of dizziness, unbalance, or non-vertiginous dizziness, generally persisting for a minimum of 3 months. This condition significantly impacts patients’ quality of life, highlighting the necessity for prompt and effective management. Vestibular rehabilitation therapy (VRT) has demonstrated efficacy in alleviating dizzy symptoms and enhancing balance. This meta-analysis seeks to assess the effectiveness of VRT in alleviating subjective dizzy symptoms and improving postural control in persons with PPPD.

**Methods:**

Two reviewers independently searched electronic databases (PubMed, EMBASE, SCOPUS, and the Cochrane Library) for pertinent studies investigating VRT for PPPD. Studies employing the Dizziness Handicap Inventory (DHI) as an assessment tool were included, provided they were extracted and presented in English from inception to March 7, 2025. Eight papers were included into this meta-analysis.

**Results:**

The aggregated weighted mean difference (WMD) indicated substantially improved outcomes for PPPD patients who underwent VRT compared to the control group in DHI-total scores (WMD = 21.84, 95% CI: [10.97, 32.71]). In subgroup analysis, DHI-total score improvements were observed in both customized VRT (WMD = 21.06, 95% CI: [5.65, 36.47]) and virtual reality-based VRT groups (WMD = 23.77, 95% CI: [8.09, 39.45]). Detailed data for DHI-Physical (DHI-P), Emotional (DHI-E), and Functional (DHI-F) scores from five trials (442 individuals) indicated a substantial reduction among PPPD patients receiving VRT (DHI-P: WMD = 17.92, 95% CI: [6.47, 29.38]; DHI-E: WMD = 10.51, 95% CI: [0.83, 20.19]; DHI-F: WMD = 15.00, 95% CI: [6.28, 23.72]).

**Conclusion:**

VRT can provide improvement for patients with PPPD, especially in DHI-total scores, DHI-P, DHI-E and DHI-F scores. Customized VRT demonstrated superior therapeutic efficacy compared with virtual reality-based VRT. However, additional randomized controlled trials are necessary to substantiate and inform the utilization of VRT in the treatment of PPPD.

## Introduction

Persistent Postural-Perceptual Dizziness (PPPD) is a persistent functional vestibular condition distinguished by particular symptoms. The symptoms predominantly consist of persistent dizziness, a sensation of instability, and non-rotational vertigo, which endure for a minimum of 3 months on the majority of days (1, 2), the symptoms may exacerbate owing to upright posture, movement (active or passive), or exposure to intricate patterns or dynamic visual stimuli. They may also be induced by disorders that result in vertigo, imbalance, dizziness, or balance-related complications (3, 4). PPPD is classified as a persistent functional vestibular illness, apart from structural or psychological disorders. It originates from a maladaptive impairment in postural regulation and central-vestibular processing, rather than a distinct anatomical anomaly in the vestibular system. In specialized dizziness clinics, PPPD constitutes the second most prevalent diagnosis, accounting for around 15–20% of cases (5). This is a recently classified illness believed to result from a prolonged maladaptation to a neuro-otological, medical, or psychological incident that precipitated vestibular symptoms. The condition entails a significant individual and healthcare burden, including reduced quality of life, prolonged sick absence, social isolation, and increased consumption of healthcare resources (6, 7). Therefore, it is essential to develop a safe and effective therapy for PPPD. The use of selective serotonin reuptake inhibitors (SSRIs) has demonstrated favorable outcomes in the management of the illness (8). Nonetheless, adverse symptoms such as nausea and exhaustion frequently lead to the cessation of therapy. Furthermore, clinical investigations have demonstrated short-term improvement by psychotherapy (9).

Vestibular rehabilitation therapy (VRT) is a program with graded exercises, consisting of eye, head, and body movements designed to stimulate the vestibular system (10). It employs customized exercises and maneuvers to promote compensation, adaptation, and habituation within the vestibular system, thereby reducing dizziness, imbalance, vertigo, and gaze instability (11). VRT includes various treatment approaches, including gaze stabilization exercises, balance training, habituation protocols, and the use of virtual reality systems. This treatment is essential for addressing the intricate vestibular dysfunctions that contribute to the persistent dizziness observed in PPPD. Its efficacy depends on regular and committed practice. VRT assists patients in overcoming maladaptive balancing habits, restoring vestibular function, and regaining everyday autonomy (12). It is a secure and efficacious non-invasive therapeutic alternative that can markedly enhance everyday functioning and quality of life (13, 14). Some studies have confirmed the effectiveness of VRT as a therapeutic intervention for individuals with PPPD (15). In a research conducted by Kundakci et al. (16), exercise-VRT offers benefits for adult patients with chronic dizziness regarding symptomatology, fall risk, balance, and emotional well-being. There has been limited research evaluating VRT outcomes in patients with PPPD (17–19), which have often exhibited a limited sample size, significant variability in caliber, and an absence of evidence-based support. Tramontano et al. (20) performed a scoping review which systematically organized existing evidence on VRT for PPPD through tabular narrative synthesis, its analytical scope was confined to descriptive statistics, precluding quantitative efficacy determinations. The present meta-analysis conducted quantitative analyses of all included studies, each of which employed the DHI as the primary outcome measure, which sought to aggregate and evaluate existing data to provide a robust evidence foundation for the efficacy of VRT and guide its therapeutic implementation.

## Materials

### Search strategy and study selection

A comprehensive literature search was conducted from inception to March 7, 2025, utilizing the MEDLINE, PubMed, Cochrane Library, Web of Science, and EMBASE databases. The subsequent search terms and keywords were interconnected by “and” or “or”: vestibular rehabilitation therapy, VRT, vestibular rehabilitation, PPPD, Persistent Postural-Perceptual Dizziness. The research was conducted in accordance with the preferred reporting items for systematic reviews and meta-analyses (PRISMA) (21)standard and was preregistered in PROSPERO (CRD420251018529, refer to the online supplemental file-PROSPERO).

## Methods

### Data extraction and study quality

#### Inclusion criteria

The search approach was limited to publicly published data and articles in English. Publications were chosen based on the subsequent criteria: Patients of any age, gender, or country diagnosed with PPPD based on clinical features and other diagnostic criteria. (2) Intervention: All patients meeting diagnostic criteria for PPPD were treated with VRT. Outcome measures (including DHI scores) were collected at baseline and upon treatment completion; (3) Outcome: The metrics employed to evaluate the relevant symptoms of PPPD encompassed vestibular symptoms, quality of life, physical capability, functional balance, and emotional well-being. The favored metric in this domain was the DHI. Diagnosis of PPPD (1): The diagnostic criteria adhere to the Bárány Society’s standards for PPPD.

#### Exclusion criteria

Two reviewers, Xiaorui Pei and Rui Ding, independently extracted data from relevant publications using data extraction forms. Discrepancies between the two reviewers were resolved by conversation with an additional author (YY Li). We excluded: (1) duplicate or irrelevant articles; (2) reviews, letters, case reports, and comments; (3) non-original research; (4) studies involving non-human subjects.

#### Data extraction

Using a standardized data collection form, two independent reviewers extracted relevant data and information from the eligible studies. This included the following: the name of the author, the year the study was published, the patient’s condition, the experimental and control groups’ treatment methods, the length of the intervention, specific outcome indicators, and other pertinent information. The effect estimates and their 95% CIs were obtained after adjusting for the greatest number of confounding factors.

#### Quality assessment

The two reviewers, Xiaorui Pei and Rui Ding, independently evaluated the quality and risk of bias of the included studies utilizing the Newcastle-Ottawa Scale (NOS) (the NOS was chosen as the quality assessment instrument because its domain-specific scoring system enables nuanced evaluation of non-randomized studies, particularly for selection bias and outcome ascertainment) (22); All disputes between the two reviewers were thoroughly examined, and moreover, a third reviewer (YY Li) was engaged to address any remaining inconsistencies and achieve a consensus.

### Statistical analysis

The meta-analysis utilized STATA version 17.0 and Review Manager software version 5.3. For continuous data, the mean ± standard deviation is shown; if the studies included utilized median and quartile values, we calculated the mean and standard deviation using the methodology of Wan et al. (23). Statistical significance was established with *p*-values < 0.05, accompanied with 95% confidence intervals. The WMD served as the metameter, while the standard deviation (SD) was utilized to assess the precision and significance of that estimate. The heterogeneity among studies was assessed utilizing Cochrane-based Q and I^2^ tests. Data with *p* < 0.05 or I^2^ > 50% were deemed to indicate statistically significant heterogeneity and were examined through the use of a random-effects model. When I^2^ < 50%, they used a fixed-effects model. Publication bias was evaluated using a funnel plot.

## Results

### Study characteristics

#### Literature search and study characteristics

**Figure 1:**
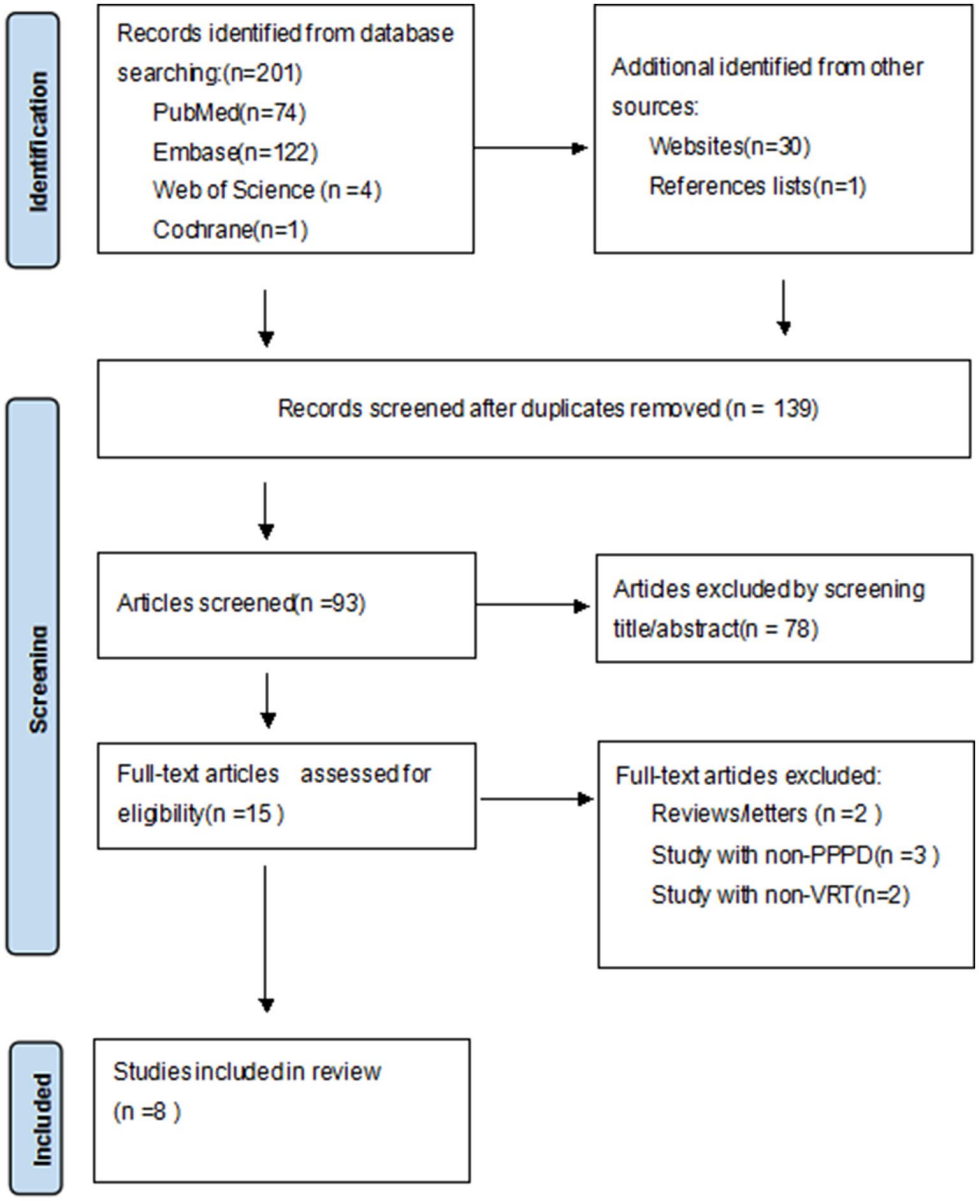
Preferred reporting item for systematic reviews and meta-analysis (PRISMA) guideline.

A total of 232 citations were identified, and only the titles and abstracts were deemed acceptable. The whole texts of possibly pertinent publications were reviewed. Table 1 enumerates the characteristics of the included studies, whereas Figure 1 illustrates the flowchart of the literature search. Following the elimination of duplicates, 93 items remained, while 78 articles were removed through the screening of titles and abstracts. Two reviews, three non-PPPD studies and two non-VRT studies were excluded. The final eight full-text papers were evaluated according to the eligibility criteria.(Figure 1).

**Table 1:** Characteristics of the included studies.

Study and year	Case/control	Intervention	Follow up (weeks)	DHI before treatment	DHI after treatment
Fujimoto 2023 (25)	12/12	Customized VRT (exercise-based and habituation exercises) once a day for 8 weeks	8	48.3 + −17.2	33.8 + −20.2
Ibrahim 2023 (34)	33/33	Customized VRT (standardized rehabilitation exercises, including Stabilization exercises, weight shifting and weight-bearing exercises and Postural control exercises, and virtual reality-based exercise) 15 min 2 times per day and for 6 weeks	6	70.5 + −12	23.3 + −9.4
Candreia 2023 (17)	23/23	virtual reality-based exercise twice per week for 30 min over a total of 2 consecutive weeks.	2	50.1 + −12.7	18.5 + −4.1
Herdman 2022 (24)	20/20	Customized VRT (walking programs and habituation, visual desensitization, static and dynamic balance exercises) 30-60 min twice a week for 16 weeks	16	65.1 + −14.7	48.8 + −19.4
Nada 2019 (33)	30/30	Customized VRT (exercises targeting gait and gaze stabilization) twice per day for 30 min for 6 weeks	6	57.8 + −16.4	36 + −14.1
Choi 2021 (26)	13/13	virtual reality-based exercise (Vestibulo-ocular reflex exercise, visual guided vestibulo-ocular reflex exercise, active head and eye exercise: the patients rotate their head and shift their gaze rapidly to catch-up to the target, optokinetic stimulation) 20 min once a week for 4 weeks.	4	42 + −9.6	26.4 + −8.9
Mempouo 2021 (35)	100/100	Customized VRT (gaze stabilization exercises and gaze stabilization exercises with visual stimulation) 20 min twice a day and Virtual reality based therapy 20 min twice a week for 8 weeks	8	58.4 + −21	50.2 + −24.3
Lin Teh 2023 (15)	30/30	Customized VRT (gaze stabilization, posture control and mobility)30 min, 3 Times a Day for 12 weeks	12	49.7 + −24	33 + −23.4

Consequently, a total of 8 papers concerning PPPD and VRT were qualitatively analyzed, followed by a meta-analysis (Table 1). The quality scores ranged from seven to nine. All contained records were deemed to be of superior quality.

#### Primary result: meta-analysis of DHI-total scores between two cohorts

**Figure 2:**
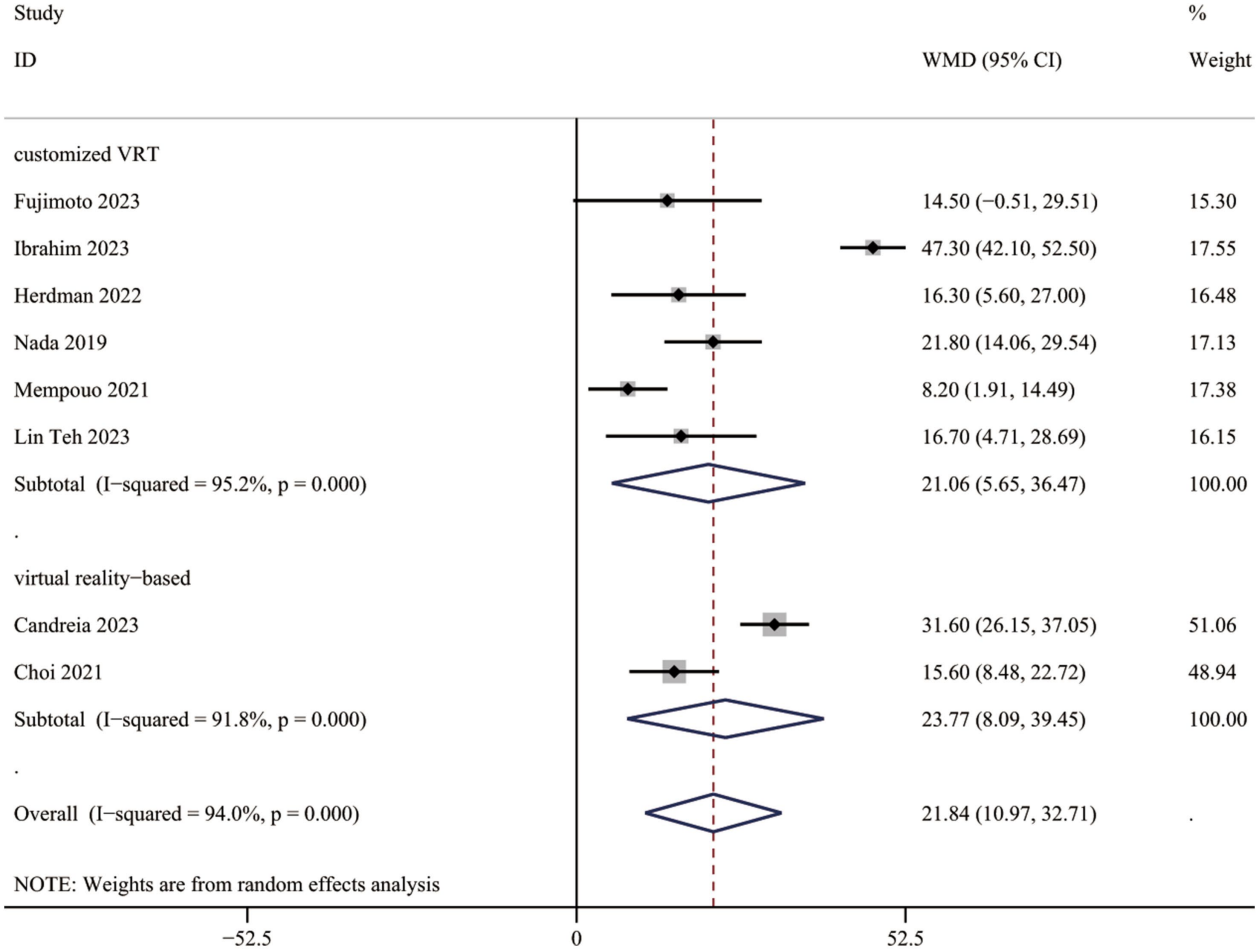
DHI-total scores between two groups.

Comprehensive data for DHI-total scores across all eight investigations (522 individuals) indicated a substantial advantage of VRT over the controls (WMD = 21.84, 95% CI: [10.97, 32.71], Figure 2).

#### Subgroup analysis in DHI-total score

Studies were grouped by VRT type: customized VRT and virtual reality-based VRT. The DHI-total score decreased significantly in PPPD patients receiving customized VRT (WMD = 21.06, 95% CI: [5.65, 36.47]) and virtual reality-based VRT (WMD = 23.77, 95% CI: [8.09, 39.45]; Figure 2).

#### Secondary result: meta-analysis of DHI functional, physical, and emotional scores comparing two cohorts

**Figure 3:**
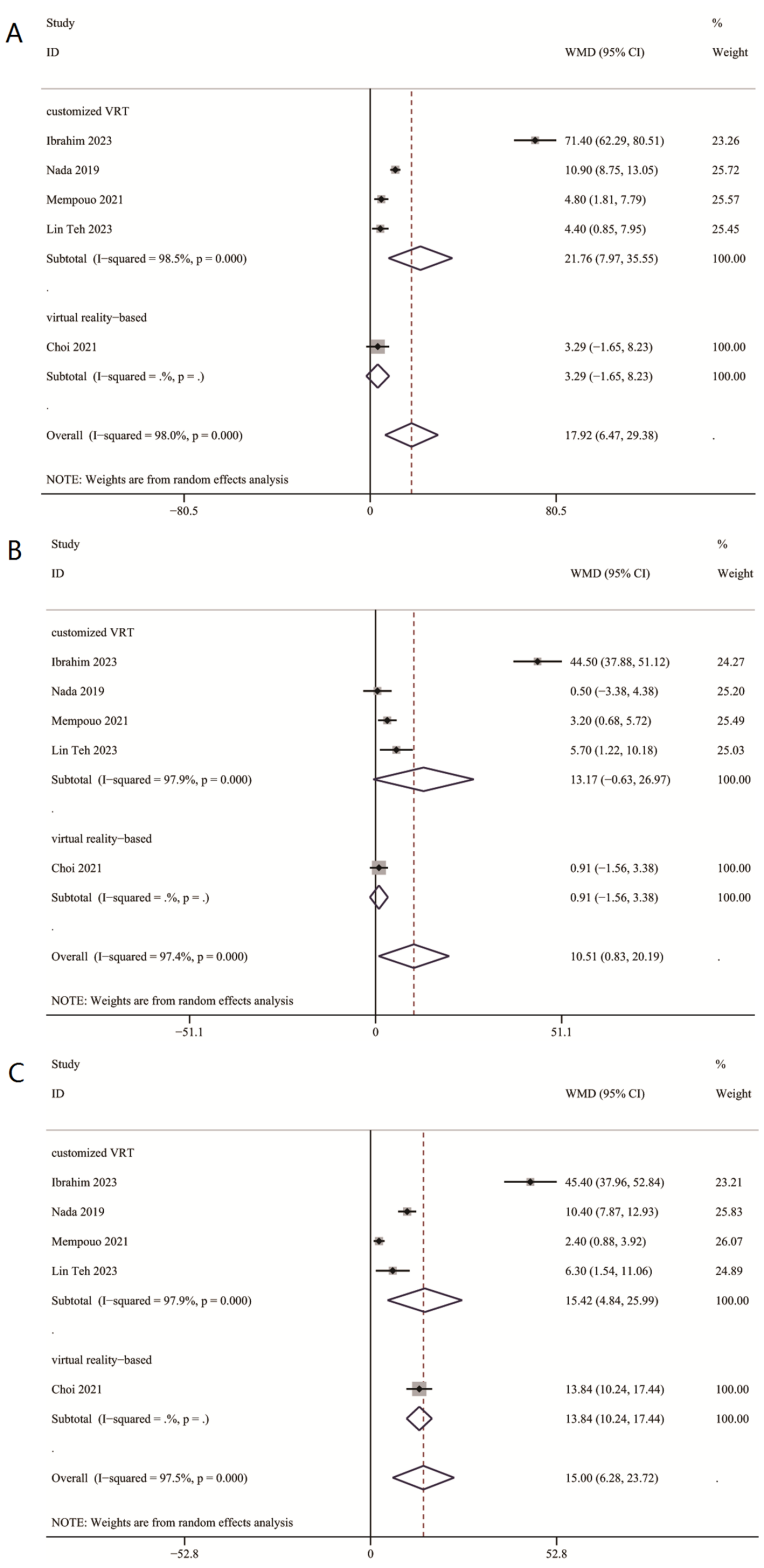
DHI-functional, physical and emotional scores between two groups. **(A)** DHI-functional scores between two groups. **(B)** DHI-physical scores between two groups. **(C)** DHI-emotional scores between two groups.

Comprehensive data for DHI-physical, emotional, and functional scores from five studies (412 participants) indicated a significant advantage of VRT over controls (DHI-P: WMD = 17.92, 95% CI: [6.47, 29.38]; DHI-E: WMD = 10.51, 95% CI: [0.83, 20.19]; DHI-F: WMD = 15.00, 95% CI: [6.28, 23.72]; Figures 3A–C).

#### Subgroup analysis in DHI functional, physical, and emotional scores

In subgroup analysis, customized VRT demonstrated significant reductions in both DHI-F (WMD = 15.42, 95%CI: [4.84, 25.99]) and DHI-P (WMD = 21.76, 95%CI: [7.97, 35.55]) scores, not in DHI-E (WMD = 13.17, 95%CI: [−0.63, 26.97]). Virtual reality-based VRT showed improvement in DHI-F scores (WMD = 13.84, 95%CI: [10.24, 17.44]), but not in DHI-E (WMD = 0.91, 95%CI: [−1.56, 3.38]) and DHI-P (WMD = 3.29, 95%CI: [−1.65, 8.23]).

#### Publication bias

**Figure 4:**
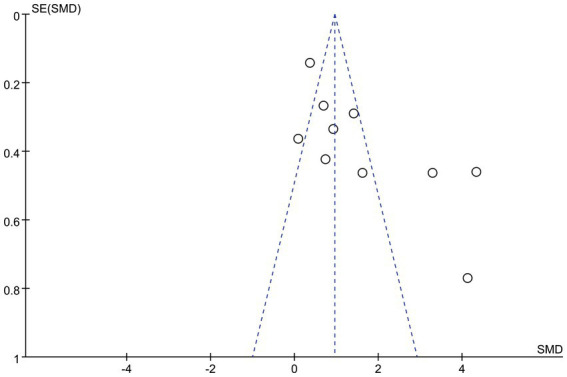
Funnel plot.

The funnel plot was basically symmetrical, and there was no evidence of publication bias (Figure 4).

## Discussion

To our knowledge, this is the first systematic review to specifically evaluate the therapeutic effects of VRT in patients with PPPD. Our findings suggest that VRT may serve as a novel and safe treatment strategy for PPPD, potentially leading to symptomatic improvement, especially customized VRT. Our meta-analysis reveals a statistically significant reduction in the total scores of the DHI questionnaire following customized VRT and virtual reality-based VRT. The current meta-analysis shows a substantial reduction across all dimensions of the DHI: physical, functional, and emotional in patients who receive VRT. In terms of functional and physical improvement, customized VRT demonstrates dual benefits, whereas virtual reality-based VRT primarily improves functional outcomes.

Certain researchers are investigating the therapeutic benefits of VRT for individuals with PPPD, but got inconsistent conclusions. Individual responses to VRT exhibit considerable variability. Some individuals may observe considerable improvement, while others may not. Herdman et al. (24) proposed that customized VRT can improve dizziness, quality of life, and gait function in PPPD. Fujimoto et al. (25) also shows that there was a significant decrease in total scores of DHI in patients receiving customized VRT, when compared with control group. Harper et al. (19) performed a randomized feasibility study and discovered that exercised-based VRT groups had a lower DHI-total than controls. Choi (26) et al. recorded significant improvements in DHI-total scores following virtual reality-based VRT among their PPPD patients. Our present meta-analysis showed that there was a significant reduction in DHI-total in patients who received VRT. Given the observed heterogeneity in the study, a subgroup analysis was performed. Subgroup analysis indicated a significant DHI-total score reduction in the customized VRT and virtual reality-based VRT groups, suggesting customized VRT and virtual reality-based VRT may as effective interventions for symptom management and prognostic improvement in PPPD patients.

Although the exact pathophysiology of PPPD is unknown, intricate relationships between the central nervous system and sensory systems are thought to be involved (14, 27). First, sensory mismatch is crucial in the pathophysiology of PPPD, since it often entails inconsistencies across sensory systems (27). This condition arises from discrepancies among visual, vestibular, and proprioceptive signals, resulting in persistent dizziness and instability (27, 28). The brain attempts to harmonize these conflicting messages, potentially resulting in enduring sensorimotor or balance problems, even in the absence of physical movement (14, 27, 28). Discrepancies may arise between visual information and vestibular signals, particularly during changes in head position or posture (27). Secondly, central sensitization significantly contributes to the pathophysiology of PPPD, when the central nervous system exhibits hypersensitivity to stimuli, leading to an amplified reaction to sensory input (29, 30). A research (31) contrasted patients with PPPD and healthy subjects administered vestibular stimulation using a motorized spinning chair. The findings indicated that PPPD patients had a diminished vestibulo-perceptual threshold, leading to increased motion sensitivity and exaggerated autonomic responses.

VRT combines adaptation, habituation and substitution, which including activities designed to enhance static and dynamic postural stability and alleviate dizziness, fostering neuroplasticity in the brain through repetitive training stimuli, and recalibrating the integration between the vestibular system and other sensory modalities, including vision and proprioception, improving visual-vestibular interactions in situations that generate conflicting sensory information (32). However, as PPPD may not have an actual vestibular deficit and instead symptoms are due to chronic hypersensitivity to motion stimuli and visual complexity, desensitization approach through habituation would be more useful (31). Furthermore, VRT can modulate the function of the vestibular nuclei and associated cerebral areas, inhibiting hyperactive neural connections. This mitigates the central nervous system’s excessive response to vestibular signals and diminishes central sensitization (30). VRT can help patients escape a cycle of maladaptive balance control, recalibrate vestibular systems, and regain independence in everyday life (14). Ultimately, VRT may improve symptoms and prognosis in individuals with PPPD.

Certain studies performed subgroup analyses according to various dimensions of the DHI. Nada et al. (33) and Choi (26) et al. discovered a notable improvement in DHI-physical and DHI-functional domains, but no improvement in the emotional domain among PPPD patients who received VRT. Ibrahim et al. (34). indicated a substantial reduction in the three areas of the DHI—physical, functional, and emotional—in patients who underwent VRT, aligning with our current meta-analysis. Improvements were observed across all domains of the DHI. Subgroup analysis (conducted due to study heterogeneity) revealed that: Customized VRT significantly reduced DHI scores in both functional and physical domains, improving symptoms and clinical function in PPPD patients; Virtual reality-based VRT only lowered DHI-functional scores. The divergence in views among studies is attributable to the pathogenic processes of PPPD. In PPPD patients, emotional elements like worry or stress may facilitate central sensitization. The interplay between central sensitization and psychosocial variables might establish a loop in which intensified emotional reactions exacerbate symptom severity (27). VRT essentially enhances vestibular function by regulating the activity of areas like the vestibular nucleus, cerebellum, and brainstem. Nonetheless, these regions have minimal overlap with brain networks linked to emotional regulation, leading to a very minor direct influence on psychological and emotional conditions (27). According to the previously indicated processes, certain researchers (26, 33) have noted emotional enhancements. Customized VRT is a patient-centered approach that targets individual clinical symptoms, underlying etiology, and functional impairments while accounting for daily living requirements (35). Through continuous therapist-guided assessment and real-time adjustments, this personalized intervention demonstrates superior efficacy in alleviating symptoms and enhancing functional outcomes in PPPD patients. In comparison, virtual reality-based VRT employs digitally simulated dynamic visual environments to facilitate central nervous system adaptation via visuo-vestibular integration. This therapeutic approach, however, is inherently limited by its dependency on predetermined algorithmic parameters and automated adjustments derived from system-generated metrics. Consequently, it demonstrates insufficient capacity for instantaneous clinical adaptation and lacks the necessary sophistication for comprehensive, pathogenesis-targeted therapeutic personalization. As the noxious stimuli is very patient specific, customized VRT protocols according to the symptoms and functional disability of the individual is encouraged (36).

The DHI was selected as the primary outcome measure in our systematic review for assessing patients with PPPD. The DHI is a well-validated, patient-reported outcome (PRO) measure comprising 25 items that quantify dizziness-related disability on a scale from 0 to 100. This tool demonstrates: (1) High reliability: Excellent internal consistency and test–retest reliability for total scores (37); (2) Clinical utility: A minimal detectable change (MDC) of 17.18 points, ensuring sensitivity to intervention effects (38); (3) Practicality: Simple administration (<10 min) and multidimensional assessment (functional, emotional, physical domains). Its standardized scoring and responsiveness to treatment outcomes make the DHI a preferred metric for evaluating dizziness management efficacy, particularly in vestibular rehabilitation protocols (37, 38).

Notwithstanding these findings, the meta-analysis had certain limitations. Primarily, most of the examined publications were studies with limited sample sizes, which are less dependable than extensive randomized controlled trials. Secondly, given the studies were limited to those published in English, we cannot eliminate the potential for publication bias, but the funnel plot is predominantly symmetrical, suggesting minimal risk. Third, there were limited shared indications for analysis. While DHI-total scores were included in the majority of the included research, the data for DHI-Physical, Emotional, and Functional scores were either unreported or inaccessible in certain studies. Finally, existing VRT studies for PPPD are few and highly variable in design, duration, and methodology. Notably, the observed heterogeneity was not resolved by subgroup analyses. Thus, systematic review findings require cautious interpretation, and treatment should be personalized. Therefore, our conclusions need to be further confirmed by additional randomized controlled trials with low heterogeneity.

## Conclusion

VRT can reduce the DHI scores in PPPD patients, including emotional, functional, and physical aspects, thereby adequately reducing symptoms and improved quality of life in subjects with PPPD. As the noxious stimuli is very patient specific, customized VRT protocols according to the symptoms and functional disability of the individual is recommended.

## Data Availability

The original contributions presented in the study are included in the article/supplementary material, further inquiries can be directed to the corresponding author.
