# Correction: Effect of vestibular rehabilitation therapy in patients with persistent postural perceptual dizziness: a systematic review and meta-analysis

**DOI:** 10.3389/fneur.2025.1719349

**Published:** 2025-11-07

**Authors:** Yanyan Li, Xiaorui Pei, Rui Ding, Ziqi Liu, Ying Xu, Zhicheng Wang, Yanmei Li, Lianhe Li

**Affiliations:** 1 Department of Neurology Ward, Chaoyang Central Hospital of China Medical University, Chaoyang, Liaoning, China; 2 Department of Laboratory of Molecular Biology, Chaoyang Central Hospital of China Medical University, Chaoyang, Liaoning, China; 3 Clinical Medicine Major, Xinhua Clinical College, Dalian University, Dalian, Liaoning, China; 4 Department of Head and Neck Surgery Ward, Chaoyang Central Hospital of China Medical University, Chaoyang, Liaoning, China

**Keywords:** vestibular rehabilitation therapy (VRT), virtual reality therapy, persistent postural-perceptual dizziness (PPPD), dizziness handicap inventory (DHI), vestibular rehabilitation

Authors “Yanyan Li and Xiaorui Pei” were erroneously omitted as equal contributing author. The original version of this article has been updated.

